# Geometric Positioning Accuracy Improvement of ZY-3 Satellite Imagery Based on Statistical Learning Theory

**DOI:** 10.3390/s18061701

**Published:** 2018-05-24

**Authors:** Niangang Jiao, Feng Wang, Hongjian You, Mudan Yang, Xinghui Yao

**Affiliations:** 1 Key Laboratory of Technology in Geo-Spatial Information Processing and Application Systems, Institute of Electronics, Chinese Academy of Sciences, Beijing 100190, China; fwang2@mail.ie.ac.cn (F.W.); hjyou@mail.ie.ac.cn (H.Y.); mdyang1993@163.com (M.Y.); Yaoxinghui1992@163.com (X.Y.); 2 Institute of Electronics, Chinese Academy of Sciences, Beijing 100190, China; 3 School of Electronic, Electrical and Communication Engineering, University of Chinese Academy of Sciences, Beijing 100049, China

**Keywords:** statistical learning theory, multi-temporal, block adjustment, rational function model (RFM), improved fast iterative shrinkage-thresholding algorithm, inherent error compensation model

## Abstract

With the increasing demand for high-resolution remote sensing images for mapping and monitoring the Earth’s environment, geometric positioning accuracy improvement plays a significant role in the image preprocessing step. Based on the statistical learning theory, we propose a new method to improve the geometric positioning accuracy without ground control points (GCPs). Multi-temporal images from the ZY-3 satellite are tested and the bias-compensated rational function model (RFM) is applied as the block adjustment model in our experiment. An easy and stable weight strategy and the fast iterative shrinkage-thresholding (FIST) algorithm which is widely used in the field of compressive sensing are improved and utilized to define the normal equation matrix and solve it. Then, the residual errors after traditional block adjustment are acquired and tested with the newly proposed inherent error compensation model based on statistical learning theory. The final results indicate that the geometric positioning accuracy of ZY-3 satellite imagery can be improved greatly with our proposed method.

## 1. Introduction

ZY-3 is the first civilian high-resolution stereo mapping satellite in China [1], and provides a tangible improvement in the monitoring capabilities for many fields, such as agriculture, forestry, geology, and so on. It has been designed for 1:50,000 scale stereo mapping requirements, which is of great significance to strengthen China’s ability to independently obtain more geospatial information. The revisit period of the ZY-3 satellite is 5 days, and so abundant high-resolution satellite (HRS) stereo image pairs can be collected over a short period. These stereo image pairs are acquired from a three-line camera (TLC) sensor on the satellite platform. The ground sampling distance (GSD) of the forward-, backward-, and nadir-directed cameras are 3.5 m, 3.5 m, and 2.1 m, respectively.

Before making use of these remote sensing images for mapping and monitoring, their geometric characteristics must be considered [2], in which the block adjustment can improve the geometric consistency and accuracy [3]. There are three types of adjustment approaches used in current research, namely bundle adjustment [4], direct georeferencing (rigorous sensor model) [5], and the rational function model (RFM) [6]. All three methods were compared in [7], and the results showed that the geometric performances were almost identical. However, considering the simplicity of implementation and standardization [8], the RFM method is widely used in the field of remote sensing image processing. During the procedure of geometric positioning accuracy improvement, a large number of well-distributed ground control points (GCPs) are usually required [9]. Due to limitations of manpower and finance, it is always difficult to acquire enough GCPs to meet the accuracy requirement [10], especially in some areas which are difficult for humans to reach, such as deserts and mountains. Many research works have proved that block adjustment with few GCPs can also achieve a high geometric positioning accuracy [11], while considering the processing mission of block adjustment for large-scale regions it may be not as good as the results in small areas. Therefore, some effective methods of block adjustment are proposed to achieve the accuracy requirement without using GCPs [6]. Nowadays, such methods can be divided into two classes: using some other known information such as the digital elevation model (DEM) [12], or taking advantage of the stereo image pairs [13]. The first case depends on the precision of the auxiliary data, while the other has a requirement of additional image data sets. In 2010, a DEM-aided block adjustment method was presented by Teo et al. [7] which is significant for improving the geometric consistency between overlapping images, and this method has been improved in [14] for nadir viewing images that constrains the elevations of tie points to improve the relative accuracy. These tie points can serve to build links between neighboring images. The relative accuracy means the relative errors between tie points which represent the same object on neighboring images. High relative accuracy of these tie points can guarantee the accuracy of image stitching. Recently, a Shuttle Radar Topography Mision(SRTM) DEM-aided block adjustment procedure was developed in [15] which was highly dependent on the DEM resolution.

Considering the characteristics of ZY-3 satellite stereo images pairs, Wang et al. [13] reported that the same accuracy can be obtained for block adjustment supported with DEM and using ZY-3 satellite stereo image pairs. Tang et al. [16] proved that the nadir image from the ZY-3 satellite can achieve a high positioning accuracy without GCPs, while using a 0.5 m vertical accuracy DEM for reference. After that, Zheng and Zhang [17] conducted experiments on multi-source of ZY-3 and GF-1 using DEM-assisted bundle block adjustment for ortho-map production. The global positioning system (GPS) is used in [18] for multi-strip long-orbit bundle block adjustment by a rigorous sensor model. The results obtained above are not always satisfactory because of the limitations of the quality of the ancillary data (e.g., DEMs). Considering that ZY-3 satellite remote sensing images can satisfy the requirements of 1:50,000 scale topographic mapping, Cao [19] undertook block adjustment of multi-temporal images of ZY-3, and the residual errors were reduced because of the redundant observation. In this way, multi-temporal images are helpful in the process of block adjustment. Moreover, multi-temporal images have been applied in many fields of image processing. For example, Gao et al. [20] presented a registration algorithm of multi-temporal images based on image geometric structural and gray information. Shaunak proposed a novel deep learning based weakly-supervised framework for urban change detection using multi-temporal polarimetric synthetic aperture radar (SAR) data in [21]. Pablo [22] introduced an automatic image orientation method with multi-temporal images, which is helpful for the use of multi-temporal images in the procedure of block adjustment.

However, methods to solve the rank-deficient normal equations have seldom been detailed in previous studies. Traditional methods such as the preconditioned conjugate gradient (PCG) algorithm [19] and the spectrum correction (SC) method [23] are time-consuming and complicated. In this way, an advanced fast iterative shrinkage-thresholding (FIST) algorithm [24] which is widely used in the field of compressive sensing is improved and detailed here to solve the singularity of the normal equation matrix. The FIST algorithm has proven to be an optimal method at the first order in algorithm complexity for solving the problem of minimizing smooth convex functions [25]. Compared with the traditional FIST algorithm, the improved FIST algorithm can make a better convergence with good efficiency [26].

Moreover, the rational function model is an approximation of the rigorous sensor model after the sensor calibration and on-orbit geometry calibration [27]. The geometric positioning errors calculated with the help of rational polynomial coefficients (RPCs) contain the systematic errors of the satellite orientation (position, altitude, camera, jitter errors, and so on) and image point errors (systematic or random errors). The image point errors are mainly random errors in the acquisition of their coordinates. Compared with systematic errors, image point errors can be reduced to a small value with an accurate acquisition method. In fact, the traditional free block adjustment method without using GCPs can reduce random errors in geometric positioning to a large extent [28]. Usually, the residual errors are distributed in a specific direction and within a certain range due to the inherent errors of sensors on the satellite platform. Based on this, the inherent error compensation model is proposed to remedy the inherent errors of the satellite platform. The inherent errors of the satellite platform are also known as systematic errors. The density-based spatial clustering of applications with noise (DBSCAN) [29] algorithm is compatible with analysing the error distribution as a popular clustering method in the field of statistical learning. Compared with traditional clustering methods such as K-means [30] and decision trees [31], the DBSCAN method can automatically classify the dataset without a specified number of categories.

The remainder of this paper is organized as follows: The mathematical details of the control point free block adjustment with the improved FIST algorithm and the inherent error compensation model are presented in Section 2. Some experiments are conducted to verify the proposed model and the results are analysed in Section 3. Finally, the conclusions of our experiments are presented in Section 4.

## 2. Methodology

**Figure 1:**
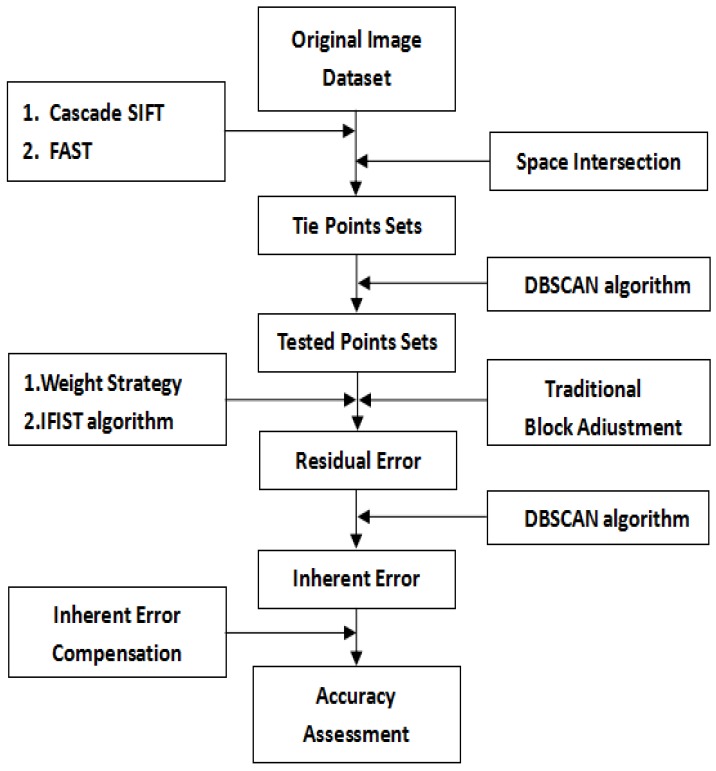
Flow chart of our proposed method. DBSCAN: density-based spatial clustering of applications with noise; FAST: features from accelerated segment test; IFIST: improved fast iterative shrinkage-thresholding.

Figure 1 shows the main steps of our proposed method. Unlike some traditional methods, our proposed method includes two stages, which are summarised as follows and described in detail below: coarse correction and accurate correction, which are comprised of the following five steps. Firstly, the tie point sets are obtained after the image registration step with the help of the cascade scale-invariant feature transform(SIFT) algorithm and features from accelerated segment test (FAST) method and the space intersection step. With the extracted dataset clustered by the DBSCAN algorithm, traditional block adjustment with the improved FIST algorithm and the proposed weight strategy are applied to improve the geometric positioning accuracy. Then, the data clustering method is applied for a second time to analyze the distribution of residual errors in order to extract the inherent satellite platform errors of our experimental dataset. Finally, the inherent error compensation model is established with the help of the clustering method, aiming to reduce the inherent errors. The main steps of our experiments are introduced in detail in the following context.

### 2.1. Cascade SIFT Method and Space Intersection Method

Initially, the tie point sets can be obtained with the help of the cascade SIFT method and FAST method. The cascade SIFT method is derived from the traditional SIFT [32] method. Since the SIFT operator is invariant with position, scale, and rotation, it is suitable to be applied for remote sensing image registration. It can be written as follows:

(1) $$\left\{\begin{array}{c} L(x,y;k\sigma )=I(x,y)*G(x,y;k\sigma ),\\ D(x,y;\sigma )=L(x,y;k\sigma )-L(x,y;\sigma ), \end{array}\right.$$

where *k* is the scale operator; I(x,y) is the original image space, and G(x,y;kσ) is a Gaussian function with a standard deviation of σ; L(x,y;σ) and D(x,y;kσ) represent the Gaussian scale space and the difference of Gaussian (DoG), respectively.

The cascade SIFT method can be divided into two parts: the large coverage adapted anisotropic Gaussian-SIFT (AAG-SIFT) algorithm and the local-SIFT algorithm. The large coverage AAG-SIFT algorithm [33] is applied to a whole remote sensing image to achieve the coarse registration. Firstly, the DoG is applied in image scale to detect most points which can represent the integral structure features of the image. Removing unstable points with low contrast in all extremal points, the final feature points in image scale are obtained. Next, the gradient of these feature points are calculated and the histogram of these gradients around detected feature points are counted. The main directions of these feature points are extracted based on the histogram. After this, the descriptors of these feature points are generated, and the random sample consensus (RANSAC) algorithm is applied to screen out the correct matching points. With all coarse correct matching points in the image space detected, the coarse registration is finished.

After the coarse registration step, a small window around the object-space coordinate is extracted with the help of the RPCs. A local SIFT matching method is utilized and accurate matching points are obtained with a local-scale DoG. With the local matching method, the matching points searching process is undertaken in the local region of the image with the interference of other unrelated regions removed. The coarse correct matching points of the same local area and the accurate matching points are combined together to ensure the accurate registration.

**Figure 2:**
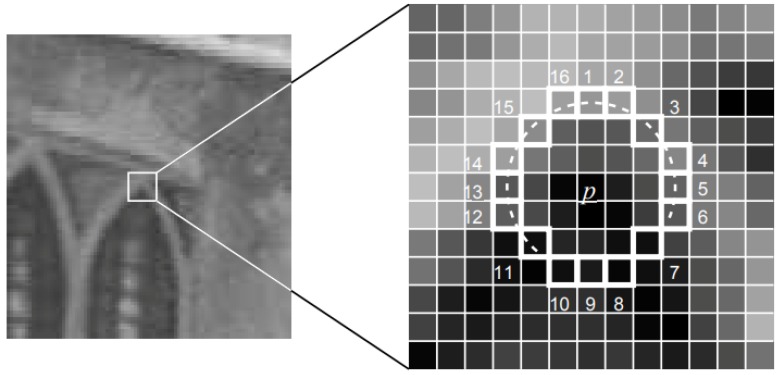
Twelve-point segment test corner detection in an image patch. The highlighted squares are the pixels used in the corner detection. The pixel at *p* is the centre of a candidate corner. The arc is indicated by the dashed line passing through 12 contiguous pixels which are brighter than *p* by more than the threshold.

After the accurate image registration, an effective corner detection method—the features from accelerated segment test (FAST) method [34]—is applied to acquire the tie point sets. A 9 × 9 window is used to strengthen the stability of the tie point matching process, as shown in Figure 2. The characteristics of the neighborhood of a tie point is calculated by the ratio as:

(2) $$\left\{\begin{array}{c} Ratio\left(k\right)=\frac{\mu (p)}{\mu (k)},fork=1,2,\ldots ,16, \end{array}\right.$$

where μ(p) represents the pixel value in central point *p* in Figure 2, and μ(k) denotes the pixel value in template *k*. For each ratio value, the similarity is measured by comparing it with a threshold as follows:

(3) $$d_{k}=\left\{\begin{array}{c} 0,for\;1/Th<Ratio(k)<Th,\\ 1,for\;Ratio(k)<Th,\\ 2,for\;Ratio(k)>1/Th, \end{array}\right.$$

where Th is a threshold value set defined by experience. We consider the searched point as a candidate tie point if more than half of the numbers of $d_{k}$ of the searched point are continuous and they all equal to 1 or 2.

After the detection process, many candidate matching points are selected, while there exist some false detections and duplicate detections. In order to solve this issue, a false point elimination method is employed. First, we assign a score function to each candidate point based on its $d_{k}$ values [35]. For a candidate matching point, the more continuous nonzero values of $d_{k}$, the higher its score. Then, a non-maximal suppression is utilized to select the best matching point. The tie point sets can be obtained accurately with this efficiency method.

**Figure 3:**
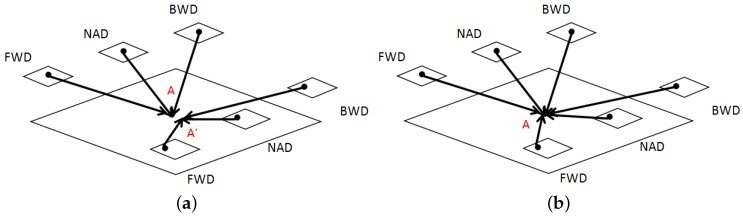
Comparison of (**a**) tie point pair and (**b**) tie point set. *A* and $A^{\prime }$ are different observations of the same object at different times. FWD, BWD, and NAD are the ZY-3 satellite images from the forward-, backward-, and nadir-directed cameras, respectively, for the same observation.

Traditionally, object-space coordinates of these tie point sets have been calculated with the aid of DEM [36] or GPS data [19], which means their accuracy is highly dependent on the precision of the auxiliary data. However, as the satellite acquires overlapping images, the space intersection method is suitable for ZY-3 stereo image pairs to derive initial values of these tie point sets. Moreover, traditional methods may cause the problem of weak convergence. Figure 3a displays an example of weak convergence. *A* and $A^{\prime }$ are different observations of the same object at different times. Due to the systematic errors of the platform and image distortion, the calculated coordinates of *A* and $A^{\prime }$ with the help of each stereo image pair usually disagree. Therefore, the multi-observation dataset provides redundant information and strong constraints which can lead to faster convergence and more accurate solutions in object-space. In this procedure, all stereo image pairs which contain the same object are used together to form overdetermined equations.

(4) $$\left[\begin{array}{ccc} \frac{\partial G_{r}^{0}}{\partial P} & \frac{\partial G_{r}^{0}}{\partial L} & \frac{\partial G_{r}^{0}}{\partial H}\\ \frac{\partial G_{c}^{0}}{\partial P} & \frac{\partial G_{c}^{0}}{\partial L} & \frac{\partial G_{c}^{0}}{\partial H}\\ \vdots & \vdots & \vdots \\ \frac{\partial G_{r}^{n}}{\partial P} & \frac{\partial G_{r}^{n}}{\partial L} & \frac{\partial G_{r}^{n}}{\partial H}\\ \frac{\partial G_{c}^{n}}{\partial P} & \frac{\partial G_{c}^{n}}{\partial L} & \frac{\partial G_{c}^{n}}{\partial H} \end{array}\right]\left[\begin{array}{c} \Delta P\\ \Delta L\\ \Delta H \end{array}\right]=\left[\begin{array}{c} l_{r}^{0}\\ l_{c}^{0}\\ \vdots \\ l_{r}^{n}\\ l_{c}^{n} \end{array}\right],$$

with

(5) $$\left\{\begin{array}{cc} G_{r} & =\frac{Num_{L}\left(P,L,H\right)}{Den_{L}\left(P,L,H\right)}\cdot Line\_Scale+Line\_Off-r,\\ G_{c} & =\frac{Num_{S}\left(P,L,H\right)}{Den_{S}\left(P,L,H\right)}\cdot Sample\_Scale+Sample\_Off-c, \end{array}\right.$$

with

(6) $$\left\{\begin{array}{c} \frac{Num_{L}\left(P,L,H\right)}{Den_{L}\left(P,L,H\right)}=\frac{a^{T}u}{b^{T}u},\\ \frac{Num_{S}\left(P,L,H\right)}{Den_{S}\left(P,L,H\right)}=\frac{c^{T}u}{d^{T}u}, \end{array}\right.$$

where $\left(G_{r}^{n},G_{c}^{n}\right)$ represent the error functions between the calculated and extracted image-space coordinates in the *n*th image; (r,c) are the extracted image-space coordinates of an object, and (P,L,H) are the object-space coordinates and (ΔP,ΔL,ΔH) are the corrections of the object-space to be solved; Line_Off,Line_Scale,Sample_Off, and Sample_Scale are the scale and translation operators of the calculated image-space coordinates with RPCs [6]; *l* is the vector of residual errors; $Num_{L},Den_{L},Num_{S}$, and $Den_{S}$ are the rational polynomial model including 80 rational polynomial coefficients (RPCs) with degree no more than three, and u=[1 *L* *P* *H* LP LH PH $L^{2}$ $P^{2}$ $H^{2}$ PLH $L^{3}$ $LP^{2}$ $LH^{2}$ $L^{2}P$ $P^{3}$ $PH^{2}$ $L^{2}H$ $P^{2}H$ $H^{3}]$, $a=[a_{0}$ $a_{1}$ … $a_{19}]$, $b=[b_{0}$ $b_{1}$ … $b_{19}]$, $c=[c_{0}$ $c_{1}$ … $c_{19}]$, and $d=[d_{0}$ $d_{1}$ … $d_{19}]$ are the rational polynomial coefficients.

To solve the overdetermined equations, the least square method (LSM) is applied. The problem of Equation (4) can be simplified as

(7) $$\left\{\begin{array}{c} Gx=l,\\ \min\{\sum_{i=1}^{n}x^{2}\}, \end{array}\right.$$

where *G* is the design matrix which consists of the partial derivatives of Gr, Gc to *P*, *L*, and *H*; and *l* is the residual errors vector between the calculated and observed image coordinates.

Through the singular value decomposition of the designed matrix *G*, the least-squares solutions can be obtained and the coordinates of the object in all images will be determined uniquely. With the help of multi-temporal images used together in the overdetermined equations, different observations will converge to the object *A* as shown in Figure 3b.

### 2.2. Data Classification and Preprocessing

The object-space coordinates of tie points are obtained through the space intersection step from the stereo image pairs. However, there are always some points with large geometric positioning errors compared with errors in other points which may be caused by errors in the image registration step or other factors. Therefore, points with large geometric positioning errors must be separated from the other data. Fortunately, this step can be regarded as a data classification problem. The object-space coordinates of the tie points are calculated from the extracted image-space coordinates and the RPCs, and the extracted object-space coordinates can be obtained from the space intersection step. Ideally, the original errors between these two values of the tie points should distribute around their extracted object-space coordinates within a certain range. Therefore, the density-based spatial clustering of application with noise (DBSCAN) method [29] based on statistical learning theory which is widely used in the field of machine learning is suitable here for data screening.

**Figure 4:**
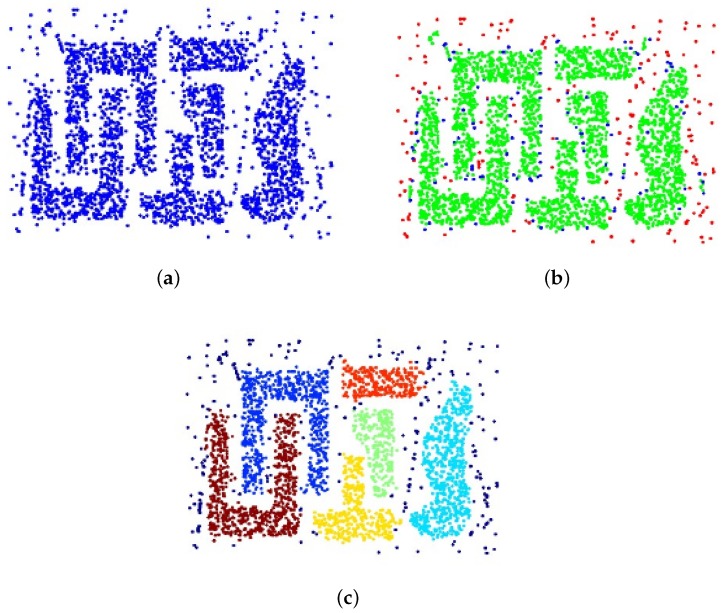
The explanation of the density-based spatial clustering of applications with noise (DBSCAN) algorithm. (**a**) is the original distribution of the dataset. (**b**) is the distribution of different type of the dataset: core (green) points, border (blue) points and noise (red) points. (**c**) is the cluster results with the DBSCAN algorithm applied to the dataset.

Supposing we have a dataset as shown in Figure 4a, and all points in this dataset can be divided into three types: core points (green points distributed inside the dense regions in Figure 4b), border points (blue points distributed at the edge of the dense regions in Figure 4b), and noise points (red points distributed in sparse regions in Figure 4b). In order to separate each type of these points with no a priori knowledge, their density distribution is important information that we can rely on. Two parameters are set before the density-based searching: Eps represents the max distance between neighborhood points in the same category and Minpts, which means the minimum number of points in one category. Searching from one point randomly in the tested dataset at first, if there are more than Minpts points in the Eps neighborhood of this point, it can be regarded as a core point and it can form a category. If the distances between points which do not belong to this category and the number of points in the formed category are smaller than Minpts, then these points are added to the formed category. If there are points which do not belong to any category, they are regarded as noise points. In this way, different categories can be formed and the noise points are separated from each category as shown in Figure 4c. An appropriate set of Eps and Minpts can achieve a satisfactory output of the classification process. Compared with other classification algorithms, the DBSCAN algorithm can detect the number of categories in the test dataset with no bias in the shape of clusters. Moreover, noise points can be quickly separated from other information in the dataset with no prior knowledge. With the help of the DBSCAN algorithm, we classify our dataset while identifying noise points to achieve a better convergence.

### 2.3. Free Block Adjustment

Because of its simplicity of implementation and standardization, the rational function model is widely used as a block adjustment model for the exterior orientation of high-resolution satellite images. It describes the relationship between image space and object space by a ratio of two cubic polynomials with 80 coefficients [6] as:

(8) $$\left\{\begin{array}{cc} X & =\frac{Num_{L}\left(P,L,H\right)}{Den_{L}\left(P,L,H\right)}=\frac{a^{T}u}{b^{T}u},\\ Y & =\frac{Num_{S}\left(P,L,H\right)}{Den_{S}\left(P,L,H\right)}=\frac{c^{T}u}{d^{T}u}, \end{array}\right.$$

where (X,Y) is the normalized image-space coordinates.

However, due to the lack of accuracy in measuring the exterior orientation elements of spaceborne sensors, the RPCs have a low precision. Therefore, an affine transformation model (AFM) is usually applied here to compensate for the bias in the RPCs to improve their precision. The AFM is defined as follows:

(9) $$\left\{\begin{array}{cc} F_{r} & =a_{0}+a_{1}\cdot Sample+a_{2}\cdot Line-r,\\ F_{c} & =b_{0}+b_{1}\cdot Sample+b_{2}\cdot Line-c, \end{array}\right.$$

where $a_{0},a_{1},a_{2},b_{0},b_{1}$, and $b_{2}$ are affine transformation coefficients, Sample and Line represent the image-space coordinates determined by RPCs, and (r,c) are the real image-space coordinates measured automatically. As a result, the error equations for the least squares solution can be derived in the form of [16]:

(10) V=At+Bs−l,

where *V* is the residual vector, *A* and *B* are the design matrices which consist of the partial derivatives of $F_{r},F_{c}$ to $a_{0},a_{1},a_{1},b_{0},b_{1},b_{2}$, and P,L,H; *t* and *s* are the correction vectors of the affine transformation parameters and object-space coordinates, respectively; *l* is the difference of the calculated and observed image point coordinates [17].

Moreover, Equation (9) applying the Gauss–Newton model can be rewritten in the matrix form as follows:

(11) $$\left[\begin{array}{cc} A^{T}PA & A^{T}PB\\ B^{T}PA & B^{T}PB \end{array}\right]\left[\begin{array}{c} t\\ s \end{array}\right]=\left[\begin{array}{c} A^{T}Pl\\ B^{T}Pl \end{array}\right],$$

where *P* is the weight matrix.

For simplicity, Equation (9) can be written as

(12) $$\left[\begin{array}{cc} U & W^{T}\\ W^{T} & V \end{array}\right]\left[\begin{array}{c} t\\ s \end{array}\right]=\left[\begin{array}{c} l_{u}\\ l_{v} \end{array}\right].$$

As a result, matrices *U* and *V* are both diagonal. *s* and *t* are dependent on each other, and usually the number of unknowns in *s* is much larger than *t*. Therefore, we use a Gauss elimination method to reduce the complexity of the unknown matrix, and the normal equation is reduced to

(13) $$\left\{\begin{array}{cc} (U-WV^{-1}W^{T})t & =l_{u}-WV^{-1}l_{v},\\ Vs & =l_{v}-W^{T}t. \end{array}\right.$$

### 2.4. Weight Strategy

In order to acquire a better convergence of the results, a good weight strategy plays an important role in the iteration step. However, researchers have seldom mentioned it in detail in their papers. With gross points removed from the experimental dataset in the data classification and preprocessing step, we can make sure that values of all tie points are valid estimations. Thus, it is appropriate to apply a stable and effective weight strategy. In our study, all observations are independent and the measurement accuracy of each element in *s* is the same. The bias in the RPCs of different images vary, which means that elements in the matrix *t* of each image share the same value. Thus, the initial weight of *s* and *t* as unity. During the iteration, the weight of *s* and *t* are updated separately under our weight strategy as follows:

(14) $$P_{s_{i}}^{m}=\frac{1}{\sigma s_{i}^{m-1}\cdot \Delta s_{i}^{m}},$$

(15) $$P_{t_{j}}^{m}=\left\{\begin{array}{cc} \frac{k^{j}}{\sigma l_{j}^{m-1}\sum_{t=1}^{k^{j}}\sqrt{\Delta x_{t}^{2}+\Delta y_{t}^{2}}} & \mathrm{for}\;a_{0}^{j},b_{0}^{j},\\ \frac{k^{j}}{\sigma l_{j}^{m-1}\sum_{t=1}^{k^{j}}\sqrt{\Delta x_{t}^{2}+\Delta y_{t}^{2}}}/H_{j} & \mathrm{for}\;a_{1}^{j},b_{1}^{j},\\ \frac{k^{j}}{\sigma l_{j}^{m-1}\sum_{t=1}^{k^{j}}\sqrt{\Delta x_{t}^{2}+\Delta y_{t}^{2}}}/W_{j} & \mathrm{for}\;a_{2}^{j},b_{2}^{j}, \end{array}\right.$$

where $P_{s_{i}}$ and $P_{t_{j}}$ represent the weights of the *i*th tie point set in *s* and the weight of the *j*th image in *t* in the *m*th iteration, respectively; $\Delta s_{i}^{m}$ and $\sigma s_{i}^{m-1}$ represent the bias and standard deviation of elements in *s*, respectively; $\Delta x_{t}$ and $\Delta y_{t}$ are the image coordinate errors of the *t*th point in the *j*th image, and $k^{j}$ is the number of image points in the *j*th image; $\sigma l_{j}^{m-1}$ is the standard deviation of the difference of all tie points between the calculated coordinates in the iteration step and coordinates extracted in the space intersection step in the *j*th image; $\left(H_{j},W_{j}\right)$ is the length and width of the *j*th image.

### 2.5. The Improved FIST Algorithm

The fast iterative shrinkage-thresholding algorithm is an advanced algorithm originating from the traditional iterative shrinkage-thresholding algorithm (ISTA). It is a popular algorithm in solving the minimization of the L1 norm problem in the compressive sensing field. Based on the algorithm proposed by Nesterov to solve the problem of minimizing smooth convex functions, the FIST algorithm has proved to be an “optimal” method at first order in algorithm complexity [25]. This method is attractive due to its simplicity and suitability for solving large-scale problems as well as achieving better convergence.

Considering a basic linear inverse problem as

(16) Res=Ax,

with an ill-conditioned coefficient matrix A, regularization methods are required to stabilize the solution, in which $l_{1}$ regularization has attracted a revival of interest and a considerable amount of attention. In this way, the linear inverse problem can be expressed as follows:

(17) $$\min_{x}\left\{F\left(x\right)={∥Ax-Res∥}^{2}+\lambda {∥x∥}_{1}\right\}=\min_{x}\left\{F\left(x\right)=f\left(x\right)+g\left(x\right)\right\},$$

with L(∇f) is the Lipschitz constant of ∇f. An approximate mapping of the problem is [26]

(18) $$prox\left(x\right)=argmin\frac{1}{2}{∥x-z∥}^{2}+\rho g\left(z\right)\left(\rho >0\right),$$

where ρ is the fixed step size within the range of $\left(0,\frac{2}{L(\nabla f)}\right)$ to guarantee the convergence of Equation (18). To solve Equation (18), the FIST algorithm consists of the following three steps:

(1) Initialization: $x_{0}$ = 0, $z_{1}$ = $x_{0}$, $t_{1}$ = 1,$\rho <\frac{2}{L(\nabla f)}$.

(2) Iteration:

(19) $$\begin{array}{c} x_{k}=prox\left(z_{k}-\nabla f\left(z_{k}\right)\right),\\ t_{k+1}=\frac{1+\sqrt{1+4*t_{k}^{2}}}{2},\\ z_{k+1}=x_{k}+\frac{t_{k}-1}{t_{k+1}}\left(x_{k}-x_{k-1}\right). \end{array}$$

(3) Estimation: if ${∥x_{k}-x_{k-1}∥}^{2}$ < ϵ, then $x_{k}$ is the expected result; else return to the iteration step. where $x_{k}$ is the unknowns vector, $z_{k}$ is a combination vector of the unknowns.

To accelerate the rate of convergence in the above iteration, an improved step size operator $\rho_{k}$ based on the Barzilai–Borwein (BB) algorithm [37] is applied, and the iteration step can be rewritten as (2) Iteration:

(20) $$\begin{array}{c} x_{k}=prox\left(z_{k}-\rho_{k}\nabla f\left(z_{k}\right)\right)T\left(b_{k}\right)=sgn\left(b_{k}\right){\left(\left|b_{k}\right|-\lambda \right)}_{+},\\ r_{k-1}=x_{k}-x_{k-1},g_{k-1}=s_{k}-s_{k-1},s_{k}=\nabla f\left(z_{k}\right),\\ \rho_{k}=\frac{r_{k-1}^{T}\cdot r_{k-1}}{r_{k-1}^{T}\cdot g_{k-1}}\left(\mathrm{if}\;\rho_{k}>\frac{1}{L},\rho_{k}=\phi \rho_{k}\right),\\ t_{k+1}=\frac{1+\sqrt{1+4*t_{k}^{2}}}{2},\\ z_{k+1}=x_{k}+\frac{t_{k}-1}{t_{k+1}}\left(x_{k}-x_{k-1}\right), \end{array}$$

where $\rho_{k}$ is the ratio of $\Delta x_{k}$ and $\Delta s_{k}$, which represents the rate of convergence; ϕ is an arithmetic factor to guarantee the convergence of the iteration. According to the Barzilai–Borwein (BB) algorithm [37], the iteration step factor can be decided by the information from the current iteration and the former one which represents the rate of convergence at $x_{k}$ .

Compared with the traditional FIST algorithm, the parameters $\rho_{k}$ cannot only optimize the iteration step of $x_{k}$, but also contribute to better convergence rates. Furthermore, the parameter $z_{k+1}$ is a specific linear combination of $x_{k-1},x_{k}$, which can significantly outperform the traditional IST algorithm and other classical gradient methods.

### 2.6. Inherent Error Compensation Model

With the random errors reduced efficiently by the above free block adjustment model, the remaining errors are mainly composed of three parts: errors due to the inaccuracy of RPCs, systematic errors, and image points errors. Errors of image points and RPCs can be constrained into a small range with an affine transformation model added in the free block adjustment procedure. Furthermore, the RPCs are an approximation of the rigorous sensor model, which means errors due to the inaccuracy of RPCs originate from the systematic errors of the satellite platform to some extent. The systematic errors include errors of the satellite orientation such as those due to position, altitude, camera, jitter, etc. Most of the systematic errors of the satellite platform can be corrected manually to a great extent. However, there will always be residual errors in the satellite orientation caused by the limitations of current technology, which is the cause of the bias between RPCs and the real sensor model. Therefore, the analysis of the distribution of the residual errors based on the statistical learning theory is an important aspect in our experiment.

Firstly, the residual errors of all tie points after block adjustment are clustered by the DBSCAN algorithm and graphed with the aid of location information. Considering the statistical property of our dataset, experimental data with few noise points are analysed and a cluster center is obtained. Since all residual errors in images from a stable sensor on the satellite platform may share the same inherent errors, all errors should have the same distribution after control point free block adjustment. Without ground control points, the actual bias between the calculated coordinates and actual coordinates is unknown. Thus, the average of the calculated coordinates contains the inherent errors of the sensor before they are removed from each point, given that the block adjustment method without GCPs does not enable their determination. Corrections based on a rigorous sensor model are helpful in restricting systematic errors of the sensor, but are not suitable for a general rational function model with RPCs. A control point free block adjustment method can result in better convergence of the computation of the correspondence between images by reducing the random errors, which means a high precision of relative positioning accuracy can be achieved. Since systematic errors exist during the whole process of block adjustment, the statistical analysis is helpful in handling them.

According to the analysis above, residual errors after control point free block adjustment are mainly the systematic errors of the sensor with small RPC errors and images point errors, which we refer to as inherent errors. Therefore, the error distributions of all tie points are consistent. Furthermore, the relationship between the relative positions of images with respect to each other are improved greatly by the above free block adjustment method. We need to improve the absolute geometric positioning accuracy. Considering that the clustering center of all residual errors are the same, an affine transform model in image-space is inefficient and not straightforward. In order to achieve accurate absolute positioning, a perspective transformation model of the object-space is added as an additional condition. The scale of errors in object-space are quite different from that in image-space, and a 1 pixel difference in image space can cause a 3 m error in the object-space. Therefore, a constraint in the object-space based on the perspective transformation model is appended with a translation model in the image-space as follows:

(21) $$\left\{\begin{array}{cc} F_{r} & =a_{0}+Sample-r,\\ F_{c} & =b_{0}+Line-c,\\ F_{P} & =k_{00}+k_{01}\cdot P_{c}+k_{02}\cdot L_{c}+k_{03}\cdot H_{c}-\bar{P},\\ F_{L} & =k_{10}+k_{11}\cdot P_{c}+k_{12}\cdot L_{c}+k_{23}\cdot H_{c}-\bar{L},\\ F_{H} & =k_{21}\cdot P_{c}+k_{21}\cdot L_{c}+k_{23}\cdot H_{c}-\bar{H}, \end{array}\right.$$

where $\left(k_{00},k_{01}\right)$ are the estimated inherent errors obtained from the former experiment of the homologous dataset in the direction of longitude and latitude, or the direction of the along-track and cross-track; $\left(\bar{P},\bar{L},\bar{H}\right)$ is the average of the calculated object-space coordinates $\left(P_{c},L_{c},H_{c}\right)$, and $\left(F_{P},F_{L},F_{H}\right)$ are the residual errors of the object-space coordinates. In order to simplify the computation, a linear transformation model of the image-space coordinates is applied as a result of the achievement of the free block adjustment. The last three lines of Equation (21) can be written in matrix form as:

(22) $$\left[\begin{array}{c} F_{P}\\ F_{L}\\ F_{H} \end{array}\right]=\left[\begin{array}{c} k_{00}\\ k_{10}\\ 0 \end{array}\right]+\left[\begin{array}{ccc} k_{01} & k_{02} & k_{03}\\ k_{11} & k_{12} & k_{13}\\ k_{21} & k_{22} & k_{23} \end{array}\right]\left[\begin{array}{c} P_{c}\\ L_{c}\\ H_{c} \end{array}\right]-\left[\begin{array}{c} \bar{P}\\ \bar{L}\\ \bar{H} \end{array}\right],$$

where the vector of $[k_{00}$ $k_{10}$ ${0]}^{T}$ is a translation coefficient in object-space obtained according to the distribution of inherent errors, which is the main compensation item of the formula. The matrix which includes the elements from $k_{01}$ to $k_{23}$ of Equation (22) are aimed to strengthen the relative relationships between images in both image-space and object-space. Equation (22) originates from the perspective transformation in the object-space as

(23) $$\left[\begin{array}{ccc} \bar{P} & \bar{L} & \bar{H} \end{array}\right]=\left[\begin{array}{ccc} P_{c} & L_{c} & H_{c} \end{array}\right]\left[\begin{array}{ccc} k_{01} & k_{02} & k_{03}\\ k_{11} & k_{12} & k_{13}\\ k_{21} & k_{22} & k_{23} \end{array}\right].$$

## 3. Experimental Results

### 3.1. Study Area and Data Set

**Figure 5:**
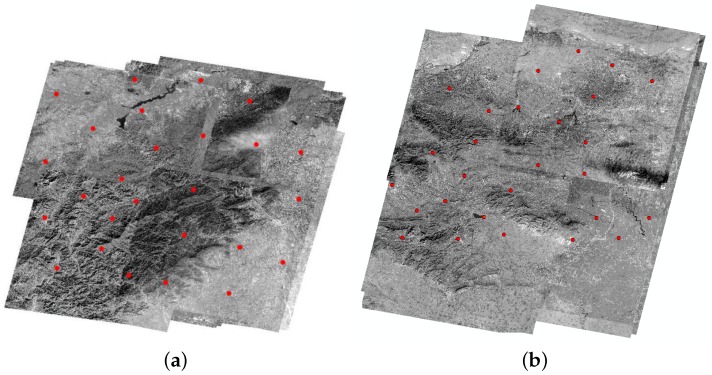
Experimental data distribution of (**a**) Beijing and (**b**) Songshan area.

In our experiment, 95 multi-observation images of Beijing and Songshan from ZY-3 were tested, covering a total area of about 35,000 km${}^{2}$ and acquired from February 2012 to January 2015, with the largest time interval being 2 years and 11 months. In the Beijing area, the elevation ranges from 30 m to 1900 m with a few mountainous areas. There were 26 tie point sets in the experimental area, 18 of which were check point sets and all 281 tie points were extracted in 35 images. The maximum number of overlapping images was 22. For the Songshan area, there were 20 groups of ZY-3 image pairs and 27 tie point sets. Twenty of them were check point sets and a total of 423 tie points were distributed in all 60 stereo images. The elevation of this area ranges from 72 m to 1200 m, with most mountains distributed throughout the area. The maximum number of overlapping images was 31. The experimental area and distribution of the tie point sets are shown in Figure 5.

### 3.2. Tie Point Sets Acquisition

**Figure 6:**
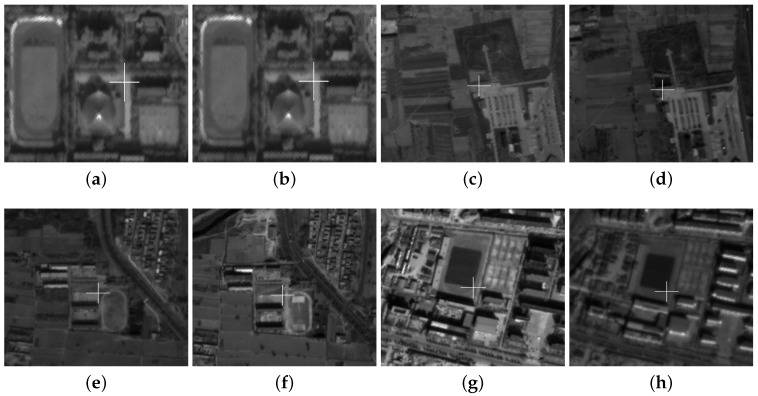
Examples of tie point sets. (**a**,**b**) are two examples of the extracted image-space points from different images in tie point set P14, where the centers of the crosses are the extracted image-space point correlated with the object in object-space. (**c**,**d**) are two examples of the extracted image-space point from different images in tie point set P22, (**e**,**f**) are two examples of the extracted image-space point from different images in tie point set P34, and (**g**,**h**) are two examples of the extracted image-space point from different images in tie point set P53.

Based on the cascade SIFT and FAST algorithms, we developed an automatic matching program to detect the potential tie points in every test image. Firstly, the large coverage scale adapted anisotropic Gaussian-SIFT(AAG-SIFT) algorithm was applied at image scale, which is helpful to find the matching areas between images quickly. Then, 300 × 300 pixel windows covering the tie points were calculated with RPCs to ensure the accuracy of the matching points during the accurate matching process. After this, the FAST method was applied in the local accurate registration regions to obtain the tie points in all images. Figure 6 shows some examples of the extracted tie point sets.

### 3.3. Control Point Free Block Adjustment

After data preprocessing, the traditional method of block adjustment was applied to the selected dataset. A stable and efficient weight strategy was applied here after the previous clustering and filtering, which means all test data is credible. To evaluate the performance of the free block adjustment method, we calculated the root mean square error(RMSE) of the absolute errors of the check points and the positioning accuracy in-plane was measured by the distance as follows

(24) $$\left\{\begin{array}{cc} \mu_{X} & =\sqrt{\frac{{\left(X_{c}-X\right)}^{2}}{n}},\\ \mu_{Y} & =\sqrt{\frac{{\left(Y_{c}-Y\right)}^{2}}{n}},\\ \mu_{P} & =\sqrt{\frac{{\left(X_{c}-X\right)}^{2}+{\left(Y_{c}-Y\right)}^{2}}{n}}, \end{array}\right.$$

where $\left(X_{c},Y_{c}\right)$ and (X,Y) represent the calculated and actual coordinates of the check points in object-space, and $\left(\mu_{X},\mu_{Y},\mu_{P}\right)$ are used for the accuracies in *X*, *Y* and the resulting vector directions.

**Figure 7:**
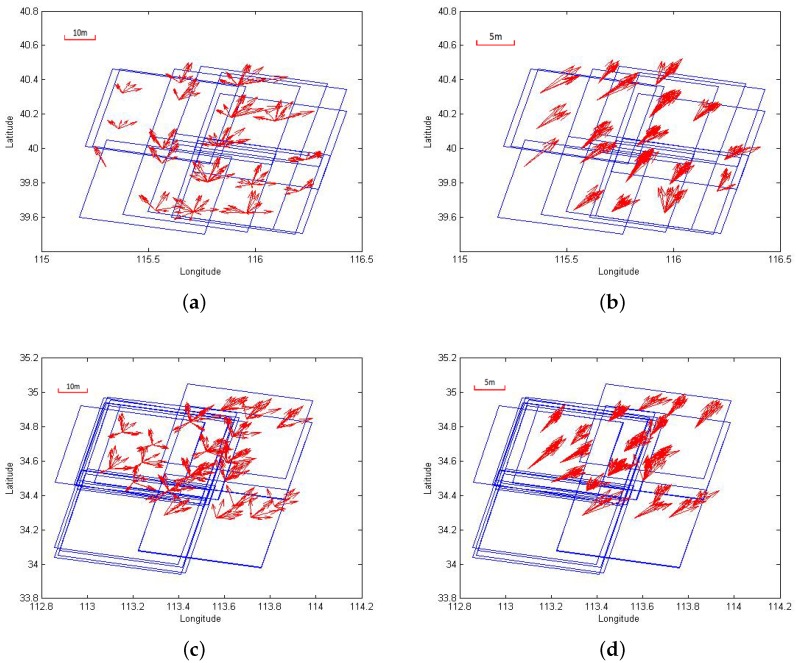
Comparison of error distribution of each check point set of (**a**,**b**) Beijing and (**c**,**d**) Songshan area. (**a**,**c**) Represent the original error distribution, while (**b**,**d**) represent the residual error distribution after block adjustment.

By the aid of the DBSCAN algorithm, the errors of all tie points were clustered together. In other words, the distribution of the absolute values of these check points indicates that almost all “noise” points in the previous dataset were removed, which contributed to a better convergence and faster computation. The error distributions of each check point set of Beijing and Songshan areas are compared in Figure 7. The directions of the arrows in the graphs indicate the directions of the error distribution, and the length represents the value of the error point. From the figure, we can see that the use of the stable weight strategy and IFIST algorithm to solve the normal equations is efficient in handling the free block adjustment. With the help of the multi-temporal dataset, the random errors of the image-space coordinates could be reduced by the multi-observation images. With the effect of the random errors in image-space reduced, the residual errors were mainly the systematic errors.

Considering the error distributions graphed in Figure 7, the absolute errors of the experimental areas were almost the same in direction and numeric value, and therefore not dependent on the geographical locations of the test datasets. As for the ZY-3 satellite, cameras of this satellite are static on the platform so that the systematic errors for all homologous data should be the same, which demonstrates consistency between the residual errors derived during the block adjustment process and the theoretical analysis. Furthermore, the error distribution converged in a certain direction, and the numerical values of the RMSE of these errors were small, as shown in Table 1.

**Figure 8:**
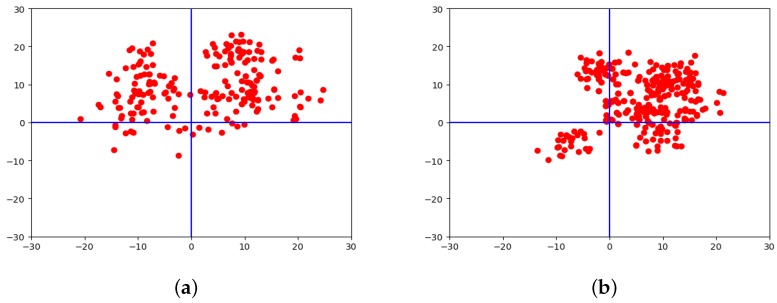

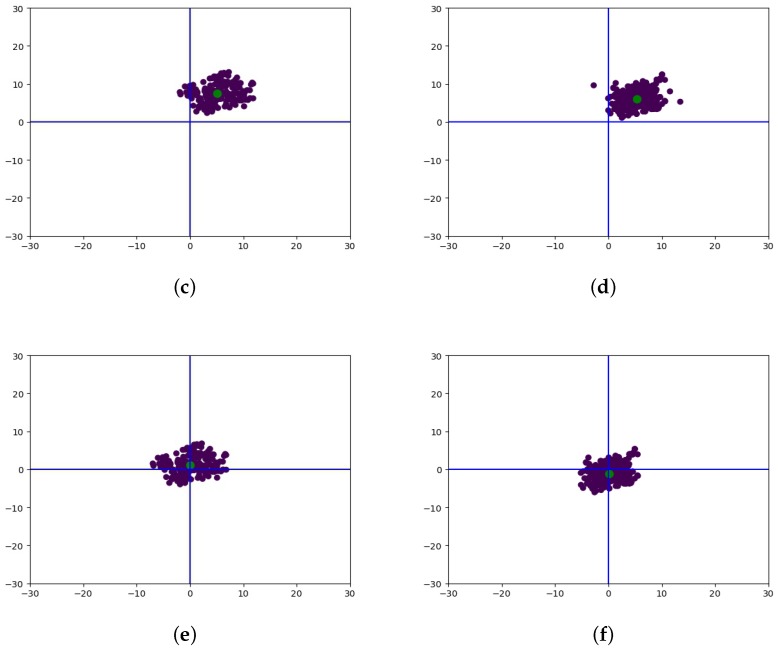
A Comparison of error distribution of all check points of Beijing and Songshan areas. (**a**,**b**) represent the original error distribution of Beijing and Songshan areas, respectively; (**c**,**d**) represent the residual error distribution after control point free block adjustment of Beijing and Songshan areas, respectively; (**e**,**f**) represent the error distribution after inherent error compensation of Beijing and Songshan areas, respectively.

The absolute errors of all check points are displayed together in Figure 8. Based on the DBSCAN theory, the green point in Figure 8c–e is the cluster center point, which shows the convergence of the block adjustment. Comparing the results in Figure 8a,c, the error distribution after the block adjustment showed an obvious convergence. While considering the error distribution of Figure 8c,d, we can see that the errors of different experimental areas converged in almost the same direction, and the cluster center was almost the same for the Songshan area as for the Beijing test area.

At the same time, a verification of the proposed weight strategy and the improved FIST method was conducted. A unit weight strategy and some traditional solutions were tested. The following four methods were applied on the Beijing test dataset: the improved fast iteration shrinkage-thresholding (IFIST) algorithm, the fast iteration shrinkage-thresholding (FIST) algorithm, the preconditioned conjugate gradient (PCG) algorithm, and the spectrum correction (SC) algorithm with a unit weight strategy and the proposed weight strategy, respectively. With the same dataset of the Beijing area and 20 times iterations, the time consumed and relative accuracy of the tie points were calculated. The relative accuracy was calculated by the mean of the quadratic sum of the difference between the calculated image-space coordinates and the average of these image-space coordinates in the same tie point set. Moreover, the absolute errors of these check points were calculated with different weight strategy and the RSME and STD of the absolute errors in the resulting vector directions of latitude and longitude were calculated to validate the stability of our proposed weight strategy. The calculation formulae of the RMSE and STD are:

(25) $$\begin{array}{c} X_{RMSE}=\sqrt{\frac{\sum_{t=1}^{N}X_{i}^{2}}{N}},\\ X_{STD}=\sqrt{\frac{\sum_{t=1}^{N}{\left(X_{i}-\bar{X}\right)}^{2}}{N}}, \end{array}$$

where $X_{i}$ is the residual error of the *i*th tie point in the resulting directions of latitude and longitude; $\bar{X}$ is the mean of all residual errors $X_{i}$, and *N* is the number of the tie points.

**Figure 9:**
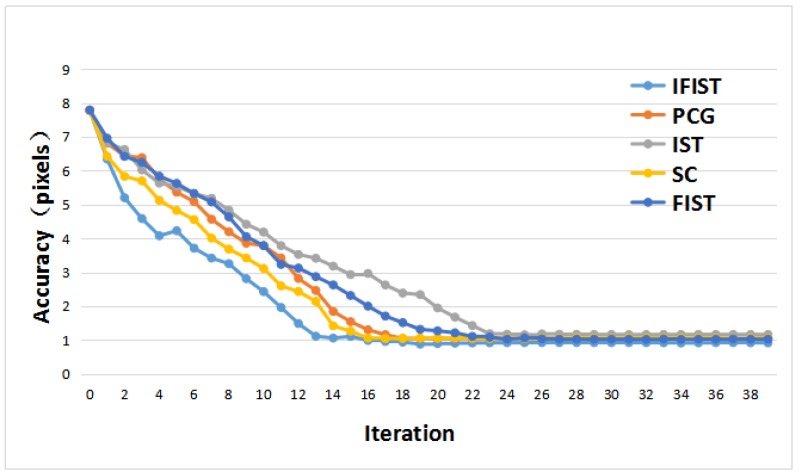
A Comparison of different methods to solve the rank-deficient equation matrix. FIST: fast iterative shrinkage-thresholding; IFIST: improved FIST; IST: iterative shrinkage-thresholding; PCG: preconditioned conjugate gradient; SC: spectrum correction.

The advantage of our proposed method which we tested with dataset of the Beijing area can be obtained by comparing the outputs of each method, which are shown in Figure 9 and Table 2. The accuracy refers to the mean value of the residual errors in image-space of each tie point, and the time consumed was conducted with the same condition of 15 iterations. Figure 9 displays the convergence of different methods with the same dataset. The IFIST algorithm was the fastest with a convergence of 13 iterations, the SC and PCG algorithms converged to stable results with almost 18 iterations, while the FIST and IST algorithms needed more than 22 iterations for their convergence. The time consumed by each method is the product of iterations and the time consumed for each iteration. Thus, the time consumed for 15 iterations and the accuracy of each method are shown in Table 2. The convergence of our proposed weight strategy of the Beijing and Songshan areas with the IFIST algorithm are shown in Table 3.

### 3.4. Inherent Error Compensation

After the data cluster step for the test dataset following the control point free block adjustment, residual errors of all tie points were clustered into the same category in which the cluster center can represent most of their characteristics. With the random errors reduced by the free block adjustment, the distribution of the residual errors indicated a systematic error in the test dataset. Considering the error distribution of different areas, the residual errors were similar while the original errors were distributed differently. With the random errors reduced, the cluster center of the residual errors are considered to be the inherent error of the sensor of ZY-3 satellite images.

**Figure 10:**
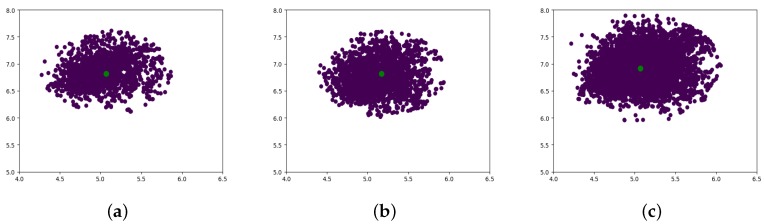
Examples of the error cluster center distribution of each combination. (**a**) The cluster centers distribution of 3416 groups of the combinations of 23 images; (**b**) The cluster centers distribution of 5468 groups of the combinations of 38 images; and (**c**) The cluster centers distribution of 8437 groups of the combinations of 54 images.The purple points are the cluster centers of each combination and the green points are the newly calculated cluster centers in all purple points.

In order to verify the theory of the inherent error compensation model, we developed a confirmatory experiment based on our test dataset, in which 23, 38, and 54 images were randomly chosen from the whole dataset to form a total of $1.8\times 10^{+27}$ combinations of images. With the free block adjustment applied to each group, the residual errors could be calculated and the cluster center of each group could be obtained with the help of DBSCAN algorithm. Then, the convergence of the results of each group were verified and all cluster centers were extracted from each group of these data sets stored in an Excel file. To reduce the time consumed, the cluster centers of 3416 groups of the combinations of 23 images, 5468 groups of the combinations of 38 images, and 8437 groups of the combinations of 54 images were randomly chosen to be graphed, and the cluster center distributions in each case are shown in Figure 10a–c. The purple points are the cluster centers of each combination and the green points are the newly calculated cluster centers in all purple points. The consistency of the residual errors indicates that all data in the test dataset shared the same characteristics. Furthermore, the coordinates of the cluster centers of the three combinations were (5.08 m, 6.82 m), (5.17 m, 6.82 m), and (5.09 m, 6.87 m) in the vector directions of longitude and latitude, respectively, which indicates that the computation of three groups converged at almost the same position. Therefore, the residual errors were considered as an inherent error of the ZY-3 satellite images based on the statistical learning theory. The inherent error compensation model is proposed to improve the geo-positioning accuracy further.

In order to acquire a more credible value of the inherent error, we put all images together to conduct the block adjustment process, and the cluster center of the residual errors of the whole dataset was located (5.13 m, 6.83 m) in the direction of longitude and latitude. Based on experiments with all data in the test dataset, this result is closer to the real value of the inherent error. As a result, the cluster center of the whole dataset was considered as an approximation of the inherent error of ZY-3 satellite imagery. The coordinates of the cluster center were utilized to propose the inherent error compensation model as $k_{00}$ and $k_{10}$ in Equation (21). With the help of the inherent compensation model, the RMSE of the geometric positioning accuracy of the test dataset was improved greatly to 4.27 m and 3.39 m in plane for the Beijing and Songshan areas, respectively. Comparison of the error distribution of the geometric positioning accuracy after the inherent error compensation model is detailed in Table 4.

**Figure 11:**
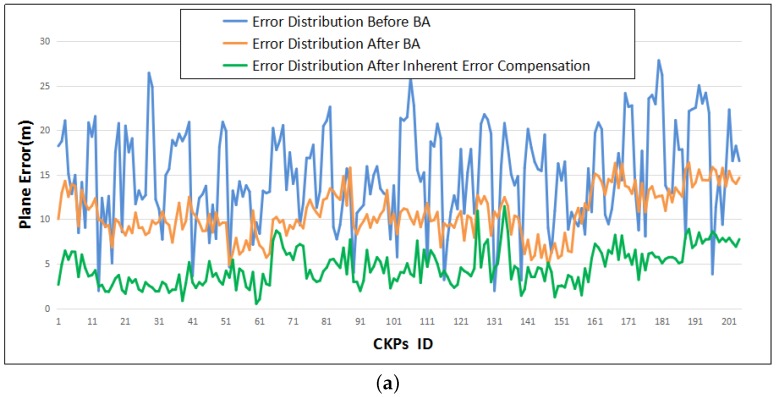

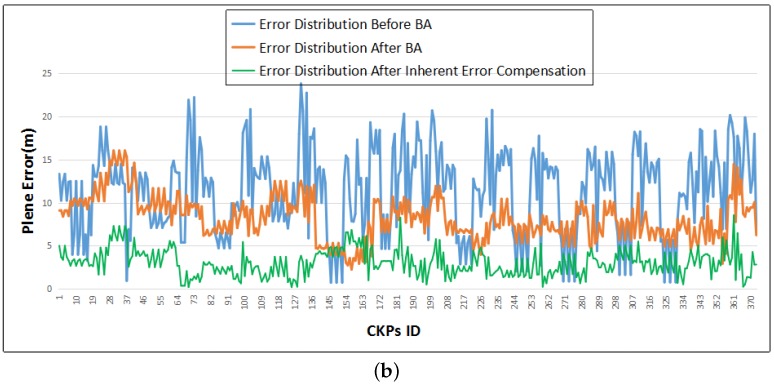
Plane error distribution of (**a**) Beijing and (**b**) Songshan areas. BA is the abbreviation of control point free block adjustment.

Furthermore, the results in Figure 8e,f are more intuitive to show the improvement of the geometric positioning accuracy of the tested datasets, and the convergence of the computations performed better compared with the results before the inherent error compensation. Figure 11 displays the improvement of the control point free block adjustment model and the inherent error compensation model. The blue line represents the error distribution of all check points in the resulting vector directions of latitude and longitude, the orange line represents the error distribution after control point free block adjustment, and the green line is the error distribution after the inherent error compensation.

## 4. Conclusions

In this paper, we put forward a new inherent error compensation model based on statistical learning theory to improve the geometric positioning accuracy of ZY-3 satellite images. Datasets of Beijing and Songshan areas were separately processed to conduct the free block adjustment together with an affine transformation model to compensate the bias of the RPCs. Simultaneously with the help of a stable and efficient weight strategy and the improved FIST algorithm which is widely used in the field of compressive sensing, the geometric positioning accuracy reduced to 9.68 m and 8.47 m in plane for the Beijing and Songshan areas. Previous studies terminated once the improvement of the affine transformation model was achieved. Therefore, we put our focus on studying the distribution of the residual errors after the free block adjustment. With the random errors reduced, the distribution of the residual errors of all check points had similar characteristics. With the aid of the DBSCAN algorithm, we could obtain a regular distribution of the residual errors by statistical analysis. Based on this, the inherent error compensation model is put forward and the geometric positioning accuracy of the test datasets was improved to 4.27 m and 3.39 m in plane for the Beijing and Songshan areas, respectively. The final experimental results showed that the residual errors of the test datasets converged to within 2 pixels in geometric positioning accuracy of ZY-3 remote sensing images. Further research of this method will be tested on remote sensing images from other platforms and multi-source datasets.

## Figures and Tables

**Table 1:** Comparison of positioning accuracy in different areas. (BA is the abbreviation of control point free block adjustment, RMSE is the root mean square error and STD represents the standard deviation).

Study Area	RMSE Before BA (m)			RMSE After BA (m)		
	Lat	Long	Plan	Lat	Long	Plan
Beijing	11.54	11.61	16.37	6.94	5.75	9.68
Songshan	8.39	9.01	12.31	5.78	2.44	8.47

**Table 2:** Comparison of different methods.

Method	IFIST	FIST	IST	PCG	SC
Time (s)	7.97	8.07	11.52	10.44	15.05
Accuracy (pixels)	1.11	2.31	2.93	1.54	1.26

**Table 3:** Comparison of residual errors with different weight strategies (m). (BA is the abbreviation of control point free block adjustment).

Weight Strategy	Error of Beijing Area				Error of Songshan Area			
	Before BA		After BA		Before BA		After BA	
	RMSE	STD	RMSE	STD	RMSE	STD	RMSE	STD
Unity	16.37	5.53	10.79	4.42	12.31	4.82	9.84	3.74
Proposed	16.37	5.53	9.68	3.09	12.31	4.82	8.47	2.57

**Table 4:** Error distribution after inherent error compensation.

Study Area	STD (m)			RMSE (m)		
	Lat	Long	Plan	Lat	Long	Plan
Beijing	3.03	2.31	2.09	3.21	2.94	4.27
Songshan	2.37	2.16	1.52	2.37	2.44	3.39

## References

[1] Xie J., Wang X. A robust autonomous star identification algorithm for ZY3 satellite. Proceedings of the First International Conference on Agro-Geoinformatics.

[2] Zhang G., De-Ren L.I., Yuan X.X., Zhang C.L. (2006). The Mapping Accuracy of Satellite Imagery Block Adjustment. J. Zhengzhou Inst. Surv. Mapp..

[3] Szeliski R., Shum H.Y. (1999). Block Adjustment Method and Apparatus for Construction of Image Mosaics. U.S. Patent.

[4] Habib A., Shin S.W., Kim K., Kim C., Bang K.I., Kim E.M., Lee D.C. (2007). Comprehensive analysis of sensor modeling alternatives for high resolution imaging satellites. Photogramm. Eng. Remote Sens..

[5] Weser T., Rottensteiner F., Willneff J., Poon J., Fraser C.S. (2008). Development and testing of a generic sensor model for pushbroom satellite imagery. Photogramm. Rec..

[6] Grodecki J., Dial G. (2003). Block adjustment of high-resolution satellite images described by rational polynomials. Photogramm. Eng. Remote Sens..

[7] Teo T.A., Chen L.C., Liu C.L., Tung Y.C., Wu W.Y. (2010). DEM-aided block adjustment for satellite images with weak convergence geometry. IEEE Trans. Geosci. Remote Sens..

[8] Dial G., Grodecki J. Block adjustment with rational polynomial camera models. Proceedings of the ASCM-ASPRS Annual Conventions.

[9] Zhang Y., Zheng M. (2012). Bundle Block Adjustment with Self-Calibration of Long Orbit CBERS-02B Imagery. ISPRS Int. Arch. Photogramm. Remote Sens. Spat. Inf. Sci..

[10] Kadota T., Takagi M. Acquisition method of ground control points for high resolution satellite imagery. Proceedings of the 23rd Asian Conference on Remote Sensing.

[11] Crespi M., Baiocchi V., De V.L., Giannone F. A new rigorous model for the orthorectification of syncronous and asyncronous high resolution imagery. Proceedings of the Earsel Symposium on New Strategies for European Remote Sensing, IUC.

[12] Chen Q., Li T., Gao X., Chen W., Wu D. Block adjustment with airborne InSAR for high-precision DEM extraction. Proceedings of the Geoscience and Remote Sensing Symposium.

[13] Wang T., Zhang G., Li D., Jiang W., Tang X., Liu X. (2014). Comparison between plane and stereo block adjustment for ZY-3 satellite images. Acta Geod. Cartogr. Sin..

[14] Zhang Y., Wan Y., Huang X., Ling X. (2016). DEM-Assisted RFM Block Adjustment of Pushbroom Nadir Viewing HRS Imagery. IEEE Trans. Geosci. Remote Sens..

[15] Chen X., Zhang B., Cen M., Guo H., Zhang T., Zhao C. (2017). SRTM DEM-Aided Mapping Satellite-1 Image Geopositioning Without Ground Control Points. IEEE Geosci. Remote Sens. Lett..

[16] Tang X., Zhou P., Zhang G., Wang X., Jiang Y., Guo L., Liu S. (2015). Verification of ZY-3 Satellite Imagery Geometric Accuracy Without Ground Control Points. IEEE Geosci. Remote Sens. Lett..

[17] Zheng M., Zhang Y. (2016). DEM-aided bundle adjustment with multisource satellite imagery: ZY-3 and GF-1 in large areas. IEEE Geosci. Remote Sens. Lett..

[18] Zhang Y., Zheng M., Xiong X., Xiong J. (2015). Multistrip bundle block adjustment of ZY-3 satellite imagery by rigorous sensor model without ground control point. IEEE Geosci. Remote Sens. Lett..

[19] Cao J. Block adjustment of zy-3 multi-temporal images without ground control points. Proceedings of the 2017 2nd International Conference on Frontiers of Sensors Technologies (ICFST).

[20] Gao X., Liu X., Yang Y., Hong L. Multi-temporal and multi-view based remote sensing image registration for ground surface objects change monitoring. Proceedings of the 2017 13th IEEE International Conference on Electronic Measurement Instruments (ICEMI).

[21] De S., Pirrone D., Bovolo F., Bruzzone L., Bhattacharya A. A novel change detection framework based on deep learning for the analysis of multi-temporal polarimetric SAR images. Proceedings of the 2017 IEEE International Geoscience and Remote Sensing Symposium (IGARSS).

[22] D’Angelo P. Automatic Orientation of large multitemporal Satellite Image Blocks. Proceedings of the International Symposium on Satellite Mapping Technology and Application.

[23] Huang D.L., Shi J.J., Lian Q., Sun L.J. (2013). Iterative algorithm based on the morbid equation. Sci. Surv. Mapp..

[24] Beck A., Teboulle M. (2009). A Fast Iterative Shrinkage-Thresholding Algorithm for Linear Inverse Problems. SIAM J. Imaging Sci..

[25] Nesterov Y.E. (1983). A method for solving the convex programming problem with convergence rate *O*(1/*k**s**p*2). Dokl. Akad. Nauk SSSR.

[26] Chen Shaoli Y.M. (2017). An Improved Fast Iterative Shrinkage-thresholding Algorithm with Variable Stepsize. Comput. Technol. Dev..

[27] Yang W.U., Liu J., Liu Z.L., Zhou J.M. (2015). Accuracy Optimization of RPC Model Based on Linear Features Control. Remote Sens. Inf..

[28] Liu C.B., Dong L.I., Tao J.H., Xue N. (2016). Direct Georeferencing of ZY-03 Imagery without(or with one)Ground Control Point. Geomat. Spat. Inf. Technol..

[29] Zhou A., Zhou S., Cao J., Fan Y., Hu Y. (2000). Approaches for scaling DBSCAN algorithm to large spatial databases. J. Comput. Sci. Technol..

[30] Hartigan J.A., Wong M.A. (1979). Algorithm AS 136: A K-Means Clustering Algorithm. J. R. Stat. Soc..

[31] Quinlan J.R. (1986). Induction of Decision Trees.

[32] Lowe D.G. (2004). Distinctive Image Features from Scale-Invariant Keypoints. Int. J. Comput. Vision.

[33] Wang F., You H., Fu X. (2015). Adapted Anisotropic Gaussian SIFT Matching Strategy for SAR Registration. IEEE Geosci. Remote Sens. Lett..

[34] Rosten E., Drummond T. Machine learning for high-speed corner detection. Proceedings of the 9th European Conference on Computer Vision.

[35] Jiao N., Kang W., Xiang Y., You H. (2017). A Novel and Fast Corner Detection Method for Sar Imagery. Int. Arch. Photogramm. Remote Sens. Spat. Inf. Sci..

[36] Quan Y., Zhang Z.H., Lim W.H., Liu W. A New Technique for Ray Tracing Point-Based Geometry. Proceedings of the 2007 International Conference on Machine Learning and Cybernetics.

[37] Hasal M., Pospisil L., Nowakova J. (2016). Barzilai-Borwein method in graph drawing algorithm based on Kamada-Kawai algorithm. American Institute of Physics Conference Series.

